# Effectiveness of a school-based educational video intervention for the prevention of technology-facilitated sexual violence among adolescent girls: a sequential explanatory mixed-methods study

**DOI:** 10.1186/s12889-026-28700-7

**Published:** 2026-08-01

**Authors:** Nurmukaromatis Saleha, Restuning Widiasih, Iqbal Pramukti, Meita Dhamayanti, Laili Rahayuwati, Binahayati Rusyidi, Suriyati

**Affiliations:** 1 Doctoral Student, Faculty of Medicine, Padjadjaran University, Jalan Ir. Soekarno No. KM. 21, Hegarmanah, Jatinangor District, Sumedang Regency, West Java 45363 Indonesia; 2 Nursing Department, Faculty of Mathematics and Natural Sciences, University of Bengkulu, Jl. Indragiri, Padang Harapan, Gading Cempaka District, Bengkulu City, Bengkulu 38225 Indonesia; 3 Department of Maternity Nursing, Faculty of Nursing, Padjadjaran University, Jl. Ir. Soekarno KM 21, Hegarmanah Sumedang, West Java 45363 Indonesia; 4 Department of Community Health Nursing, Faculty of Nursing, Universitas Padjadjaran, Sumedang, West Java Indonesia; 5 Department of Child Health, Faculty of Medicine, Universitas Padjadjaran, Sumedang, West Java Indonesia; 6 Social Welfare Department and the Research Center for Gender and Children Studies, Universitas Padjadjaran, Sumedang, West Java Indonesia; 7 Midwifery Study Program, Faculty of Mathematics and Natural Sciences, University of Bengkulu, Jl. Indragiri, Padang Harapan, Gading Cempaka District, Bengkulu City, Bengkulu 38225 Indonesia

**Keywords:** Adolescent girls, Technology-facilitated sexual violence, Sexual violence prevention, Video-based education, School-based intervention, School health promotion, Mixed methods

## Abstract

**Background:**

Technology-facilitated sexual violence (TFSV) threatens adolescent health and safety, yet prevention content remains insufficiently addressed in routine school-based education. This study evaluated a school-based digital TFSV prevention programme and explored adolescents’ experiences and perceptions of its implementation.

**Methods:**

A sequential explanatory mixed-methods study was conducted in two public senior high schools in Bengkulu City, Indonesia. The quantitative phase used a quasi-experimental pre-test–post-test non-equivalent control group design involving 69 female adolescents (control, *n* = 33; intervention, *n* = 36). Both groups received the standard school-based programme; the intervention group additionally received six educational videos over approximately five weeks through classroom sessions, Zoom reinforcement, and repeated Instagram access. Knowledge, awareness, and attitudes were analysed using Wilcoxon signed-rank and Mann–Whitney U tests, with Holm adjustment for post-test comparisons. The qualitative phase comprised a post-intervention focus group discussion with seven participants and was analysed using reflexive thematic analysis.

**Results:**

Post-test knowledge was higher in the intervention group than in the control group (U = 375.000, adjusted *p* = 0.009, r = 0.36), as was awareness (U = 418.000, adjusted *p* = 0.044, r = 0.28). No significant between-group difference was found for attitude (U = 516.000, adjusted *p* = 0.331, r = 0.12); attitude increased significantly within both groups. Qualitatively, participants described gaps in formal TFSV education, viewed audiovisual delivery as relevant and engaging, identified perceived relevance, family support, and trust in school personnel as influences on engagement and help-seeking, and recommended integration into routine school structures with interactive delivery. Integration of both strands indicated that these experiences helped explain the gains in knowledge and awareness, while contextualising the absence of an intervention-specific attitudinal effect.

**Conclusion:**

The digital TFSV prevention programme was associated with higher short-term knowledge and awareness than the standard school-based programme, but no additional effect on attitude was demonstrated. Relevant and engaging audiovisual education may strengthen adolescents’ recognition of TFSV risks; sustained prevention may require repeated reinforcement, family engagement, trusted school-based support, and integration into routine school health promotion. Further multicentre studies with baseline-adjusted analyses and longer follow-up are warranted.

**Supplementary Information:**

The online version contains supplementary material available at https://doi.org/10.1186/s12889-026-28700-7.

## Background

Sexual and reproductive health is a core component of women’s health and an essential part of the global development agenda. Sustainable Development Goal (SDG) 3 calls for universal access to sexual and reproductive health services, information, and education, while SDG 5 emphasizes the elimination of all forms of violence against women and girls [1]. Despite these commitments, sexual violence remains a persistent public health concern, and in the digital era its spectrum has expanded to include technology-facilitated sexual violence (TFSV) as part of technology-facilitated gender-based violence. This issue is particularly important during adolescence, as digital technologies increasingly shape how young people communicate, form relationships, and negotiate social identities. Although digital environments provide opportunities for communication, learning, and participation, they also create new vulnerabilities to harassment, exploitation, and sexual violence [2–4]. Recent evidence shows that forms of TFSV, including online sexual harassment, image-based sexual abuse, and online child sexual exploitation and abuse, are associated with substantial psychological, emotional, and social harms among adolescents and young people [5, 6].

For the purposes of this study, technology-facilitated sexual violence (TFSV) was operationally defined as any unwanted, coercive, exploitative, or harmful sexual act, communication, request, threat, or misuse of sexual content in which digital technologies or online platforms are used as the medium, tool, or context for initiating, facilitating, documenting, amplifying, or threatening sexual harm. This definition includes both online-only sexual harms and digitally facilitated harms that may escalate into offline sexual abuse. In the context of this school-based prevention programme, TFSV included online sexual harassment, unwanted sexual messages or sexualised comments, online grooming, sexting-related pressure or coercion, requests for intimate images, non-consensual recording or sharing of intimate/sexual content, threats to distribute intimate content, sextortion, cyberstalking, and digitally mediated sexual intimidation. This operationalisation was used to guide the development of the educational video content, questionnaire domains, and qualitative exploration of adolescents’ knowledge, awareness, and attitudes toward TFSV prevention. The study did not assess direct victimisation experience; instead, it evaluated preventive outcomes related to adolescents’ capacity to recognise, avoid, respond to, and seek help for TFSV-related risks.

In Indonesia, TFSV and online gender-based violence have become increasingly documented concerns. In Bengkulu City, however, local reports do not yet clearly disaggregate technology-facilitated sexual violence from broader sexual violence data. Previous local evidence, including student survey data and our prior qualitative studies, indicated that online gender-based violence and digital safety risks were relevant concerns among adolescents, while schools, families, and community health services had limited structured prevention responses [7–9]. Bengkulu City was therefore selected as a locally relevant and under-documented setting for a school-based TFSV prevention intervention.

Previous school-based prevention programmes have shown benefits in improving children’s and adolescents’ knowledge of sexual abuse prevention, online safety, consent, sexual health, and protective behaviours, although evidence on sustained behavioural change and reduction of actual violence remains limited [3, 10, 11] Related interventions, including comprehensive sexuality education, sexual harassment prevention, online safety education, and web-based media literacy programmes, have used school-based or digital formats to strengthen sexual health knowledge, communication, and gender-related attitudes [12–14]. However, most have focused on child sexual abuse, sexual harassment, dating violence, online safety, or broader sexual health, rather than specifically addressing technology-facilitated sexual violence as a gendered and digitally mediated risk in adolescents’ everyday online interactions.

The present intervention extends previous work by focusing specifically on technology-facilitated sexual violence among adolescent girls and integrating sexting-related risk, online grooming, online harassment, digital safety, legal literacy, sexual and reproductive rights, self-esteem, boundary-setting, and help-seeking. It was developed from local qualitative findings in the Indonesian school context, delivered through classroom viewing, Zoom reinforcement, and repeated Instagram access, and validated by a multidisciplinary expert panel. Thus, the programme was designed not only to improve knowledge, but also to strengthen awareness and initiate protective attitudes within a school-feasible health promotion model.

The development of the intervention was theoretically informed by Pender’s Health Promotion Model (HPM) [15, 16]. In our previous exploratory qualitative studies, the interview and focus group discussion guides were developed using HPM as a sensitising framework to explore individual characteristics and experiences, behaviour-specific cognitions and affect, interpersonal influences, situational influences, and health-promoting behavioural outcomes related to TFSV prevention. These prior findings indicated that adolescents needed prevention education that was concrete, relatable, visually engaging, and closely connected to their everyday digital experiences. They also suggested that video-based media could be an acceptable and engaging educational format for adolescent girls. Therefore, the present intervention was developed by integrating HPM-informed exploratory findings, evidence from a narrative literature review, and input from multidisciplinary expert reviewers.

The nursing discipline plays a central role in this intervention, particularly through the lens of school health nursing, which integrates health promotion with a holistic and trauma-informed care approach. This nursing perspective guided the content development of the six educational videos, ensuring that technology-facilitated sexual violence (TFSV) prevention messages are delivered in an adaptive manner that avoids retraumatization among female adolescents. Grounded in this professional framework, the intervention conceptualization incorporates rigorous content validation from pediatric and community health nursing experts to ensure its developmental appropriateness and clinical accuracy [17–19]. In the Bengkulu school context, School Health Units are commonly supported by health personnel, including community nurses, who contribute to school-based health promotion. Although the present intervention was delivered by the research team, the educational videos were designed as practical tools that can support nurses and school health personnel in delivering preventive education on technology-facilitated sexual violence.

Building on this earlier work, the present study represents the next phase of research aimed at developing and evaluating a more applicable school-based preventive intervention. Educational strategies for adolescents should be aligned with the characteristics of the digital generation, who are highly familiar with audiovisual media and often respond more strongly to concrete, relevant, and experience-based visual narratives [20]. In this context, video-based education can offer advantages over conventional counseling by depicting risky situations more vividly, enhancing emotional engagement [12, 21]. Videos can thus facilitate the understanding of protective messages, such as recognizing sexually abusive behavior, setting boundaries, resisting pressure, maintaining digital safety, and seeking help [3].

Although video-based, web-based, and media-literacy sexual health education for adolescents has been widely studied and shown to improve sexual health knowledge, communication, and related attitudes, evidence remains limited in the Indonesian school context on interventions that specifically address technology-facilitated sexual violence. In particular, few studies have directly compared structured video-based TFSV prevention education with conventional counselling among school-aged adolescents in Indonesia [9, 22]. Therefore, this study aimed to: (1) compare the effectiveness of video-based education and conventional counselling in improving adolescents’ knowledge, awareness, and attitudes toward TFSV prevention; and (2) explore adolescents’ experiences, engagement, acceptability, and perceptions of the school-based digital video intervention.

## Methods

### Study design

This study used a sequential explanatory mixed-methods design. The quantitative phase employed a quasi-experimental pre-test–post-test non-equivalent control group design, followed by a qualitative phase using a post-intervention focus group discussion to explain participants’ experiences and perceptions of the intervention. Reporting of the quantitative component was guided by the TREND checklist for non-randomised evaluations of behavioural and public health interventions.

The study was conducted from January to March 2026 in two public senior high schools in Bengkulu City, Indonesia. The two schools were comparable in terms of urban location, student academic profile, and A-level school accreditation. The study was prospectively submitted to and registered within the Universitas Padjadjaran Research Ethics Committee system before data collection. Ethical approval was obtained from the Health Research Ethics Committee of Universitas Padjadjaran (Registration No. 2502050157; Approval No. 271/UN6.KEP/EC/2025). This institutional ethics system includes protocol review, amendment procedures, continuing/final reporting, and adverse event reporting. Before enrolment, all participants and their parents or legal guardians received oral and written information in Indonesian about the study objectives, procedures, potential risks and benefits, confidentiality, voluntary participation, and the right to withdraw at any time. Written informed consent was obtained from the parents or legal guardians of all participants, including those aged 16 and 17 years. In addition, assent/consent was obtained from all adolescent participants before their involvement in the study.

### Participants: inclusion and exclusion criteria

Eligible participants were female students aged 15–18 years in grades 10 or 11 who had access to digital media and provided assent/consent, with written parental or guardian consent. Final-year students were excluded because of academic commitments. Students with known mental health problems, identified through school coordination or disclosed during recruitment, were also excluded as an ethical precaution due to the sensitive nature of TFSV-related content and the potential for psychological discomfort or distress. The final analysed sample included participants aged 15–17 years.

### Sample size calculation

The sample size was determined a priori using G*Power software for a two-group mean difference test. The calculation was based on a previous comparable study by Boulton et al., with an effect size of d = 0.85, a significance level of α = 0.05, and statistical power of 0.95. The minimum required sample size was 31 participants per group. To anticipate possible attrition, the sample size was increased by approximately 30%, resulting in a target recruitment of 40 participants per group, or 80 participants in total. During implementation, 69 participants completed the study and were included in the final analysis, consisting of 33 participants in the control group and 36 participants in the intervention group.

### Recruitment and group allocation

Participants were recruited in coordination with the vice principal for student affairs at each school. Eligible participants were female students aged 15–18 years, enrolled in grades 10 or 11, had access to digital media, and agreed to participate with parental or guardian consent when required. Students in their final year and those with mental health problems were excluded.

Because randomisation was not feasible in the school setting, group allocation was conducted at the school level. One school was assigned as the control group and the other as the intervention group. This approach was used to reduce the risk of contamination between participants. Initially, 40 students were recruited for each group. After attrition, the final analysis included 33 participants in the control group and 36 participants in the intervention group. Seven participants in the control group did not complete the study due to absence during the intervention session, while four participants in the intervention group withdrew or did not complete the intervention activities.

### Intervention and comparison condition

The intervention was implemented within a five-week period according to a pre-specified schedule developed in coordination with the participating school. The five-week period referred to the overall implementation window from the baseline assessment to the post-intervention assessment, rather than to five consecutive weeks of face-to-face or synchronous sessions. Classroom video viewing and Zoom reinforcement sessions were conducted on scheduled dates that accommodated the school timetable, while the educational videos remained accessible through the study Instagram account throughout the intervention period to support repeated exposure. Videos 1 and 2 were introduced in the classroom on January 30 and reinforced through Zoom on January 31. Video 3 was delivered in the classroom on February 6 and reinforced through Zoom on February 7. Videos 4, 5, and 6 were delivered in the classroom on February 13 and reinforced through Zoom on February 14. The videos remained available on the study Instagram account during the five-week intervention period.

The intervention group received the same standard programme plus a structured digital video-based educational intervention. The intervention was delivered over approximately five weeks through classroom viewing, scheduled Zoom reinforcement, and repeated access through Instagram. The intervention consisted of six educational videos developed from prior qualitative findings [8, 9] The video topics included sexting and online grooming, cyber sexual safety, personal and interpersonal drivers of TFSV, sexual health education, digital literacy, and legal literacy. Videos 1 and 2 were introduced in class on January 30 and reinforced through Zoom on January 31. Video 3 was delivered in class on February 6 and reinforced through Zoom on February 7. Videos 4, 5, and 6 were delivered in class on February 13 and reinforced through Zoom on February 14. The videos were also uploaded to the study Instagram account to allow repeated access.

The intervention was delivered by the research team in coordination with the participating school. Although school nurses were not the primary implementers in this study, the video materials were developed as structured educational tools that could be used by community nurses and School Health Unit personnel in future school-based TFSV prevention activities.

The content validity of the videos was assessed using the Content Validity Index by seven experts in paediatric nursing, community nursing, public health, information technology, educational technology, psychology, and law [23, 24]. The S-CVI/Ave values for the six videos ranged from 0.96 to 1.00, indicating excellent content validity. An overview of the educational video content is presented in Table 1.

**Table 1:** Intervention video components and targeted outcomes

Video	Duration	Focus	Targeted outcome
1	5 min 34 s	Sexting and online grooming: drama	Improved knowledge of TSFV risk factors
2	7 min 24 s	Cyber sexual safety awareness: animation	Increased awareness of online sexual safety
3	28 min 31 Sect. (3 parts)	Personal and interpersonal drivers of TSFV: podcast	Strengthened self-esteem and reduced motivation for sexting
4	18 min 27 Sect. (2 parts)	Sexual health education	Improved understanding of sexual health as self-protection
5	5 min	Digital literacy	Improved digital literacy for TSFV prevention
6	10 min	Legal literacy	Improved legal literacy and awareness of sexual and reproductive rights

### Outcome measures and variables

The primary outcome variables were knowledge, awareness, and attitudes related to technology-facilitated sexual violence (TFSV) prevention. These outcomes were measured at two time points: before the intervention (pre-test) and after the intervention (post-test). Knowledge was measured using a Guttman-scale questionnaire, while awareness and attitudes were measured using Likert-scale questionnaires. Higher scores indicated better knowledge, greater awareness, and more supportive attitudes toward TFSV prevention. The knowledge questionnaire used a Guttman scale, while the awareness and attitude questionnaires used Likert scales. The instruments were reviewed by four experts in community nursing, public health, education, and law. Pilot testing was conducted among 30 adolescent girls with characteristics comparable to the study population. The knowledge questionnaire showed item validity coefficients ranging from 0.391 to 0.552 and a reliability coefficient of 0.652. Although this value was considered modest internal consistency for an early-stage educational intervention study using a newly developed knowledge instrument, it was below the conventional threshold of 0.70 and is therefore acknowledged as a measurement limitation. The awareness questionnaire showed item validity coefficients ranging from 0.604 to 0.857, with one item revised linguistically because of its conceptual importance; the final reliability coefficient was 0.850. The attitude questionnaire showed item validity coefficients ranging from 0.562 to 0.896 and a reliability coefficient of 0.889. The English-language version of the questionnaire was provided as Supplementary File 1.

The main independent variable was group allocation, consisting of the intervention group and the control group. The intervention group received the standard school-based programme plus the structured digital video-based educational intervention, while the control group received the standard school-based programme only. Time of measurement, namely pre-test and post-test, was used to assess within-group changes over time.

Baseline participant characteristics included grade, age, living arrangement, and daily mobile phone use. Daily mobile phone use was measured through a self-reported demographic item asking participants to estimate their average duration of mobile phone use per day. Responses were categorised into three groups: 1–3 h, 3–5 h, and more than 5 h per day. This variable was used to describe participants’ baseline digital exposure and was not treated as an intervention outcome.

### Quantitative data collection and analysis

Quantitative data were collected before and after the intervention using structured questionnaires. Baseline participant characteristics included grade, age, living arrangement, and daily mobile phone use. Baseline differences between groups were assessed using the chi-square test or Fisher’s exact test, as appropriate.

Normality was assessed using the Shapiro–Wilk test, and homogeneity of variance was assessed using Levene’s test. Because the outcome variables were not normally distributed and some post-test variables did not meet the assumption of homogeneity, non-parametric tests were used. Within-group pre-test and post-test changes were analysed using the Wilcoxon signed-rank test. Between-group differences at pre-test and post-test were analysed using the Mann–Whitney U test. Data were presented as median and interquartile range. Statistical significance was set at *p* < 0.05.

Quantitative data were collected using pre- and post-intervention questionnaires assessing knowledge, awareness, and attitudes related to technology-facilitated sexual violence (TFSV) prevention. The questionnaires were developed specifically for this study and had not been previously published elsewhere. Their development was informed by relevant literature and prior exploratory qualitative findings from interviews/FGDs. The English-language version of the questionnaires has been uploaded as Supplementary File 1 and is cited in the main manuscript.

The instruments underwent content validation by four experts in community nursing, public health, education, and law. Pilot testing was subsequently conducted among 30 adolescent girls with characteristics comparable to those of the study population.

### Qualitative data collection and analysis

A post-intervention qualitative evaluation was conducted to explain the quantitative findings and explore participants’ experiences, engagement, acceptability, perceived effects, and recommendations for programme implementation. Participants were purposively selected from the intervention group to capture variation in post-intervention scores, attendance patterns, and levels of programme engagement. Ten students were invited, of whom seven attended the focus group discussion (FGD). The selected participants included students with differing levels of participation, including those who had not attended all intervention sessions, to obtain a broader range of perspectives on the programme’s relevance, acceptability, perceived benefits, and implementation barriers.

The FGD was conducted on February 16, 2026, lasted approximately one hour, and took place in a private room to ensure participants’ privacy and comfort. The discussion was moderated by the principal researcher and conducted in Indonesian, the language shared by the moderator and participants. A semi-structured discussion guide covered five areas: experiences of participating in the programme, appropriateness and relevance of the educational content, perceived effects of the intervention, acceptability of the delivery format and technology, and recommendations for programme improvement and future implementation.

The data were analysed using Braun and Clarke’s reflexive thematic analysis, an interpretive approach used to identify patterns of shared meaning across the dataset. The analysis followed six recursive phases: familiarisation with the data, generation of initial codes, development of candidate themes, review of themes in relation to the coded extracts and the dataset as a whole, refinement and naming of themes, and production of the final analytic narrative [25, 26]. Themes were developed around central organising concepts rather than as descriptive summaries of the discussion topics. In accordance with reflexive thematic analysis, coding and theme development were understood as active interpretive processes shaped by the researchers’ engagement with the data.

The principal researcher contributed to the development and delivery of the intervention and also moderated the FGD. This dual role provided contextual familiarity but may have influenced the questions asked, participants’ willingness to express criticism, and the interpretation of favourable accounts. The qualitative findings were therefore treated as participants’ perceptions of the programme rather than as independent evidence of intervention effectiveness. Candidate themes and interpretations were reviewed through research-team discussions, with particular attention to critical and divergent accounts, including limited engagement, perceived low relevance, scheduling constraints, and lack of trust in school-based support. Themes were subsequently refined to ensure that their scope and interpretation remained proportionate to the available data and supporting quotations.

The interview guide was developed by the researcher and included the following: (1) Experience in implementing educational programs, (2) Appropriateness of materials (content), (3) Perceived impact (indications of change), (4) Acceptability and technology design, and (5) Recommendations for implementation (for model revision). Of the ten invited participants, seven attended the FGD. The discussion was held on Monday, February 16, starting at 11:00 a.m. and lasting approximately one hour. To ensure the privacy and comfort of participants, the session was conducted in a private VIP room at a restaurant. The FGD was moderated by the principal researcher. The discussion was conducted in Indonesian, which is shared by the researcher and participants. Participants were selected based on their active participation in all intervention sessions.

## Results

A total of 69 adolescent girls were included in the final analysis, consisting of 33 participants in the control group and 36 in the intervention group. Although 40 participants were initially recruited for each group, seven participants in the control group did not complete the study due to absence during the intervention session, while four participants in the intervention group dropped out prematurely due to refusal or incomplete participation. Educational videos were watched in class and during scheduled Zoom meetings. Participants were also encouraged to follow the researcher’s Instagram account, which contained educational videos. Not all female students participated in the Zoom meeting session. Similarly, on Instagram, female students only watched but showed little interest in following or commenting. The evaluation focused on changes in knowledge, awareness, and attitudes regarding TFSV prevention, as well as participants’ experiences with the intervention. Participants’ lack of engagement in the Zoom and Instagram sessions was evaluated qualitatively. The characteristics are shown in Table 2.

**Table 2:** Baseline characteristics of participants in the control and intervention groups

Characteristic	Control group (*n* = 33)	Intervention group (*n* = 36)	Total (*N* = 69)	*p*-value
Grade, n (%)				1.000
Grade 10	23 (69.7)	25 (69.4)	48 (69.6)	
Grade 11	10 (30.3)	11 (30.6)	21 (30.4)	
Age (years), n (%)				0.518
15	16 (48.5)	14 (38.9)	30 (43.5)	
16	12 (36.4)	18 (50.0)	30 (43.5)	
17	5 (15.2)	4 (11.1)	9 (13.0)	
Living arrangement, n (%)				0.105
With biological parents	26 (78.8)	34 (94.4)	60 (87.0)	
With extended family	6 (18.2)	1 (2.8)	7 (10.1)	
Living alone/boarding	1 (3.0)	1 (2.8)	2 (2.9)	
Daily mobile phone use, n (%)				0.847
1–3 h	8 (24.2)	7 (19.4)	15 (21.7)	
3–5 h	11 (33.3)	14 (38.9)	25 (36.2)	
> 5 h	14 (42.4)	15 (41.7)	29 (42.0)	

Values are presented as n (%). Baseline differences between groups were tested using the chi-square test or Fisher’s exact test, as appropriate. Statistical significance was defined as *p* < 0.05

At baseline, the control and intervention groups were comparable across all measured participant characteristics. Most participants in both groups were in Grade 10 (control: 69.7%; intervention: 69.4%; *p* = 1.000), and most were aged 15–16 years (*p* = 0.518). Most participants lived with their biological parents, although this proportion was higher in the intervention group (94.4%) than in the control group (78.8%); however, the difference was not statistically significant (*p* = 0.105). Daily mobile phone use was also similar between groups, with the largest proportion in both groups reporting more than 5 h of use per day (control: 42.4%; intervention: 41.7%; *p* = 0.847). Overall, no statistically significant baseline differences were identified between the control and intervention groups.

### Quantitative findings

Normality testing using the Shapiro–Wilk test indicated that the outcome variables were not normally distributed, and Levene’s test showed that some post-test variables did not meet the assumption of homogeneity. Therefore, non-parametric analyses were used. Within-group changes from pre-test to post-test were examined using the Wilcoxon signed-rank test (Table 3), and between-group differences were assessed using the Mann–Whitney U test (Table 4).

**Table 3:** Within-group comparison of pre-test and post-test scores using Wilcoxon signed-rank test

Outcome	Pre-test median (IQR)	Post-test median (IQR)	Z	*p*-value	Effect size, *r*	Interpretation
Group Control						
Knowledge	9.0 (8.00–10.00)	9.0 (8.00–10.00)	-1.683	0.092	0.29	Small
Awareness	48.0 (45.5–49.5)	49.0 (47.0–50.0)	-1.595	0.111	0.28	Small
Attitude	47.0 (45.5–48.0)	49.0 (47.5–50.0)	-2.359	0.018	0.41	Moderate
Group Intervention						
Knowledge	9.0 (8.25-10.0)	10.0 (9.25-10.0)	-3.247	0.001	0.54	Large
Awareness	48.0 (45.0–50.0)	50.0 (48.0–50.0)	-4.114	< 0.001	0.69	Large
Attitude	48.0 (45.0–50.0)	49.0 (48.0–50.0)	-2.046	0.041	0.34	Moderate

Within-group analysis showed no statistically significant changes in knowledge (* Z=-1.683*, * p=0.092*, * r=0.29*) or awareness (* Z=-1.595*, * p=0.111*, * r=0.28*) in the control group. However, attitude scores increased significantly, with a moderate effect size (* Z=-2.359*, * p=0.018*, * r=0.41*). In the intervention group, statistically significant increases were observed in knowledge, with a large effect size (* Z=-3.247*, * p=0.001*, * r=0.54*); awareness, with a large effect size (* Z=-4.114*, * p<0.001*, * r=0.69*); and attitude, with a moderate effect size (* Z=-2.046*, * p=0.041*, * r=0.34*). Nevertheless, because attitude increased significantly in both groups and the post-test between-group difference was not statistically significant, the findings do not demonstrate an additional effect of the digital intervention on attitude.

**Table 4:** Between-group comparison of knowledge, awareness, and attitude scores at pre-test and post-test using the Mann–Whitney U test

Outcome	Time point	Control group, Median (IQR)	Intervention group, Median (IQR)	U	Z	*p*-value
Knowledge	Pre-test	9 (8–10)	9 (8.25–10)	461.500	-1.669	0.095
	Post-test	9 (8–10)	10 (9.25–10)	375.000	-2.983	0.003
Awareness	Pre-test	48 (45–49)	48 (45–50)	581.500	-0.152	0.879
	Post-test	49 (47–50)	50 (48–50)	418.000	-2.291	0.022
Attitude	Pre-test	47 (45–48)	48 (45–50)	515.500	-0.956	0.339
	Post-test	49 (47–50)	49 (48–50)	516.000	-0.972	0.331

Outcome	Time point	Raw *p*	Holm-adjusted *p*	*R*	Statistical interpretation	
Knowledge	Pre-test	0.095	—	0.20	Descriptive baseline difference	
	Post-test	0.003	0.009	0.36	Significant after Holm adjustment; higher scores in the intervention group	
Awareness	Pre-test	0.879	—	0.02	Descriptive baseline difference	
	Post-test	0.022	0.044	0.28	Significant after Holm adjustment; higher scores in the intervention group	
Attitude	Pre-test	0.339	—	0.12	Descriptive baseline difference	
	Post-test	0.331	0.331	0.12	Not statistically significant	

Post-test knowledge scores were significantly higher in the intervention group than in the control group after Holm adjustment (* U=375.000*, * Z=-2.983*, adjusted * p=0.009*, * r=0.36*), representing a moderate effect. Post-test awareness scores were also higher in the intervention group (* U=418.000*, * Z=-2.291*, adjusted * p=0.044*, * r=0.28*), although the effect was small. No significant post-test difference was found for attitude (* U=516.000*, * Z=-0.972*, adjusted * p=0.331*, * r=0.12*). Baseline comparisons were treated descriptively and showed negligible-to-small effect sizes.

Taken together, the post-test between-group comparisons showed higher knowledge and awareness scores in the intervention group after adjustment for multiple comparisons. A different pattern was observed for attitude. Attitude scores increased significantly within both the control group (* Z=-2.359*, * p=0.018*, * r=0.41*) and the intervention group (* Z=-2.046*, * p=0.041*, * r=0.34*). However, the post-test difference between groups was not statistically significant (* U=516.000*, * Z=-0.972*, Holm-adjusted * p=0.331*, * r=0.12*). Therefore, the findings do not demonstrate an additional short-term effect of the digital intervention on attitude beyond the standard school-based programme.

### Qualitative findings

The thematic analysis generated four overarching themes: (1) gaps in formal sexual health literacy created a need for empowering TFSV education; (2) the digital intervention enhanced adolescents’ understanding, engagement, and protective awareness; (3) Engagement and help-seeking were shaped by perceived relevance, family support, and trust in school personnel; and (4) Programme sustainability required integration into school structures and interactive delivery. The participants in this FGD were Au (16 years old), N (15 years old), V (15 years old), Ar (15 years old), C (15 years old), D (15 years old), and S (17 years old).

#### Theme 1. Gaps in formal sexual health literacy created a need for empowering TFSV education

Participants felt that schools were not providing sufficient education on technology-based sexual violence. The program is perceived not merely as additional material, but as an intervention that fills a critical gap in adolescent sexual health literacy. This theme highlights an educational gap that makes the program feel relevant and empowering. Here are some participant statements:

“…this really broadens our horizons because at school there isn’t… There isn’t any material like this taught to us…” C, 15 years old.

“…this is rarely discussed by people, so it broadens our horizons.” D, 15 years old.

Education as a guest/additional curriculum is perceived as disrupting learning time.

“The teacher will be angry, right, ma’am? But I’d rather schedule more time for the subject.” Au, 15 years old.

“And also, only a few people who follow this mother’s program are rarely discussed by people, so this broadens our insight. This, so not everyone may know about this material.” C, 15 years old.

This situation also suggests that guest curriculum sexual health material is highly dependent on student preferences and the availability of time. Based on our observations, the material in standard sexual violence prevention interventions is a repetition of what students learned in elementary school. Therefore, it does not align with students’ current developmental stage as late adolescents.

#### Theme 2. The digital intervention enhanced adolescents’ understanding, engagement, and protective awareness

Digital interventions not only enhance conceptual understanding but also make the material more engaging, easier to grasp, and closely tied to everyday experiences. Supporting statements:

“I think all the videos are interesting… some are dramas, and the podcasts answer questions we might be wondering about too…” D, 15 years old.

“…the ‘Grooming’ segment with Maya’s boyfriend—from that, we can see the real-world impacts of TFSV…” Ar, 15 years old.

“…N also learned something new… and…also gave us a message to help us stay safe in the digital world…” N, 15 years old.

Audiovisual formats such as dramas, podcasts, and the character “Maya” boost engagement while fostering protective awareness, including the ability to recognize risks, impacts, and the importance of self-protection in the digital space.

#### Theme 3. Engagement and help-seeking were shaped by perceived relevance, family support, and trust in school personnel

Participants described variation in engagement with the programme according to how relevant or important they considered the topic. Engagement was also reinforced by family involvement, as one participant reported that her mother learned from the material and subsequently provided advice. In relation to help-seeking, participants identified limited comfort and trust in school guidance counsellors as a potential barrier to disclosure. They also emphasised that an appropriate first response to a peer experiencing TFSV should involve listening without judgement. Taken together, these accounts indicate that programme engagement and help-seeking were influenced by perceived relevance, family reinforcement, and the availability of trusted and non-judgmental support. This is evident in the following quotes:

“...it depends on the individual... they consider it less important...” Au, 16 years old.

“So my Mom also learns new things… she always gives advice to N…” N, 15 years old.

“Maybe many people don’t talk to the guidance counselor because they aren’t comfortable yet…” V, 15 years old.

“The first step is definitely to listen to their story… and not judge them either.” D, 15 years old.

#### Theme 4. Programme sustainability required integration into school structures and interactive delivery

Participants did not view the programme as a stand-alone activity. They suggested that it should be incorporated into existing school structures, with guidance and counselling identified as the most relevant entry point. Participants also preferred informal discussion and visually engaging content over text-heavy materials. One participant suggested that the topic could be relevant across several subjects; however, this was treated as an individual implementation suggestion rather than as evidence of an established cross-curricular model. Overall, the findings indicate that sustainability may depend on embedding the programme within routine school activities and retaining an interactive, dialogic, and visually engaging format. Supporting quotes:

“I think the subject related to that is Guidance and Counseling, ma’am…”. “For me, Ms., a casual discussion like this is actually nice…” “…if they’re given articles, they usually don’t read them…” Au, 16 years old.

“…lessons like this actually almost apply to all subjects…” “…Most people just like things that entertain them; they just want things that catch their interest, you know…” S, 17 years old.

Taken together, these findings suggest that the digital TFSV prevention education programme was more effective than the standard school-based programme in improving knowledge and awareness related to TFSV prevention.

### Integration of quantitative and qualitative findings

The quantitative and qualitative findings were complementary primarily in relation to knowledge and awareness. Quantitatively, the intervention group showed higher post-test knowledge and awareness scores than the control group after adjustment for multiple comparisons, whereas no significant post-test between-group difference was found for attitude. These findings indicate that the intervention was most strongly associated with cognitive understanding and recognition of TFSV-related risks.

The qualitative findings helped explain the higher post-test knowledge scores. Participants described TFSV as a topic that was rarely addressed in routine school-based education and perceived the programme content as new, relevant, and applicable to their everyday digital experiences. These accounts suggest that the intervention may have expanded participants’ understanding by addressing gaps in formal sexual health and digital safety education.

The qualitative data also provided explanatory insight into the awareness findings. Participants reported that videos, podcasts, and dramatized scenarios made the material clearer, more engaging, and easier to relate to real-life digital interactions. These formats appeared to support participants’ ability to recognise warning signs, understand potential consequences, and appreciate the importance of self-protection in online environments. Thus, the audiovisual and contextually relevant delivery may have contributed to the higher awareness scores observed in the intervention group.

A different interpretation is required for attitude. Attitude scores increased significantly in both the control and intervention groups, and the post-test difference between groups was not statistically significant. Therefore, the qualitative accounts concerning empathy, non-judgmental responses, disclosure, trust, and school support should not be interpreted as evidence of an intervention-specific improvement in attitude. Rather, these findings provide contextual insight into the interpersonal and institutional conditions that may shape attitudes and help-seeking. The concentration of attitude scores near the upper end of the scale may also have limited the ability of the measure to detect additional change.

Overall, the integrated findings suggest that relevant content and engaging audiovisual delivery may support adolescents’ knowledge and awareness of TFSV prevention. The qualitative findings further identified perceived relevance, family reinforcement, trust in school personnel, and interactive school-based delivery as important implementation considerations. Future implementation should therefore combine digital educational materials with facilitated discussion, repeated reinforcement, family engagement, and trusted school-based support and referral pathways.

## Discussion

This study examined the effectiveness of a digital TFSV prevention education programme in improving adolescents’ knowledge, awareness, and attitudes related to the prevention of technology-facilitated sexual violence (TFSV). Overall, adjusted post-test comparisons showed higher knowledge and awareness scores in the intervention group, whereas no significant between-group difference was demonstrated for attitude. Although attitude scores increased within the intervention group, a comparable improvement was also observed in the control group; therefore, this change cannot be attributed specifically to the digital intervention. Quantitative analysis demonstrated significant within-group improvements in knowledge, awareness, and attitude among students in the intervention group, whereas between-group post-test differences were significant for knowledge and awareness but not for attitude. These findings suggest that the intervention was particularly effective in strengthening cognitive and risk-recognition outcomes, while attitudinal change appeared to be more gradual and context-dependent.

Baseline characteristics indicated that the control and intervention groups were comparable prior to the intervention. No statistically significant differences were found between groups in grade, age, living arrangement, boyfriend status, or daily mobile phone use. This baseline similarity is important in the context of TFSV prevention research, as these characteristics may influence adolescents’ exposure to digital interactions, access to online content, and vulnerability to online risks. The groups were broadly comparable in the measured individual-level baseline characteristics, including grade, age, living arrangement, and daily mobile phone use. However, this comparability does not eliminate potential confounding. Because only one school represented each study condition, unmeasured school-level characteristics, including school climate, teacher involvement, counselling practices, peer norms, institutional support, and prior educational exposure, could not be separated from intervention allocation. The post-test differences should therefore be interpreted cautiously as post-intervention group differences rather than definitive causal effects of the digital programme.

The significant increase in knowledge is likely due to the intervention’s ability to address gaps in formal school-based education [27, 28]. Qualitative findings indicate that TFSV-related content was perceived as rarely covered in routine learning, and therefore the program was experienced not as supplementary material, but as an empowering resource that introduced new, relevant, and practically meaningful knowledge about digital sexual violence, its forms, and consequences. At the same time, because the program was delivered as a supplementary activity, access depended on time availability, school schedules, and student interest and may not reach all students. This suggests the need for more systematic integration into formal school learning to strengthen adolescent sexual health literacy [29–33].

Furthermore, sexual health education should be delivered continuously through a developmentally appropriate and sequential approach, rather than as a one-time intervention. Previous research suggests that effective school-based sexual health curricula depend on sufficient exposure to lesson content, while broader evidence on comprehensive sexuality education supports the value of age-appropriate progression across developmental stages. In this way, earlier learning can reinforce subsequent material and support the gradual development of more complex knowledge, attitudes, and protective skills [29, 34, 35] The importance of integrating sexual health education into the formal curriculum is further underscored by the limitations of relying on social media alone. Adolescents’ exposure to health information on social media is shaped by platform algorithms, browsing behaviour, and credibility cues, and young people may still struggle to assess the reliability of what they encounter online. Consequently, educational content produced by official institutions may not be consistently visible or trusted, limiting the reach of social media-based learning when used in isolation [20, 36, 37].

The improvement in awareness can be interpreted in light of the intervention’s delivery format. Participants consistently described the materials as clear, concrete, and easy to relate to real-life situations. The use of dramatized videos, podcast-style discussion, and interactive audiovisual elements appeared to increase engagement and facilitate recognition of risky situations, personal boundaries, and vulnerabilities in digital interactions [38]. Awareness in the context of TFSV prevention involves more than factual understanding; it also requires the ability to identify warning signs and interpret potentially harmful situations [39, 40]. The qualitative findings suggest that the intervention supported this process by making the content personally meaningful and emotionally engaging. Prior research indicates that adolescents respond more positively to sexual health messages that are personalized, relatable, interactive, and emotionally validating, while video-based and participatory approaches can further enhance engagement and learning [41, 42].

A different pattern was observed for attitude. Attitude scores increased significantly within both the control and intervention groups, while the post-test difference between groups was not statistically significant. Therefore, the within-group improvement in the intervention group should not be interpreted as evidence of an intervention-specific effect. Because both groups received the standard school-based programme, the observed improvement may reflect the influence of this shared educational exposure, repeated measurement, maturation, or other contextual influences operating across the study period. In addition, attitude scores were already concentrated near the upper end of the scale at baseline and post-test, leaving limited room for further measurable improvement. This possible ceiling effect may have reduced the sensitivity of the instrument to detect additional intervention-related change. Overall, the findings do not demonstrate an additional short-term effect of the digital intervention on attitude beyond the standard programme.

Attitudes toward TFSV prevention may also be less responsive to short-term educational exposure than knowledge and risk recognition [43]. Such attitudes are shaped by interpersonal trust, peer norms, family communication, stigma, perceptions of victims, and confidence in school reporting and support mechanisms. The qualitative findings similarly showed that participants continued to experience concerns regarding confidentiality, disclosure, and trust in school personnel [44–47]. Consequently, more sustained changes in attitude may require repeated reinforcement, facilitated discussion, opportunities to practise responses to realistic digital situations, and stronger support from families, peers, teachers, counsellors, and health professionals [44–46, 48–51].

The mixed-methods integration strengthens this interpretation. The quantitative findings showed measurable gains in knowledge and awareness, while the qualitative findings explained how those gains were achieved. Participants described the intervention as filling a gap in formal education, using delivery methods that were engaging and adolescent-friendly, and encouraging reflection on self-protection in digital spaces. At the same time, the qualitative results clarified why attitudinal change was less pronounced at the between-group level: adolescents’ attitudes toward TFSV are shaped not only by educational input but also by broader relational and institutional contexts. Trust in adults, family openness, peer support, and confidence in school-based reporting mechanisms all appeared to influence how participants interpreted risk and whether they felt able to act on what they had learned [52–58].

An important finding from the qualitative data was that participation and responsiveness were shaped by factors beyond the intervention itself. Students described variation in motivation, perceived relevance, competing activities, and willingness to engage with the content. Some participants suggested that peers who did not continue in the programme may have considered the topic unimportant, preferred to seek information independently, or lacked sufficient interest. Others emphasized the influence of family members, including the role of parents in reinforcing learning, encouraging caution, and shaping disclosure patterns [59]. These findings indicate that trust in adults, family openness, peer support, and confidence in school-based reporting and support systems appear to shape how adolescents interpret risk and whether they feel able to act on what they have learned [48, 57, 60–64]. Prior research similarly suggests that disclosure and help-seeking are strongly influenced by relational trust, family and school support, peer networks, and adolescents’ awareness of and confidence in formal support mechanisms [65, 66].

The findings also highlight the importance of safe and trusted support systems. Participants often viewed peers as the most accessible first line of support, but also acknowledged their own limitations in responding effectively when a case occurred nearby. School counselling services were not always perceived as fully trusted spaces, largely because of concerns about confidentiality and unfamiliarity. This is important because improved awareness alone may not lead to timely help-seeking if adolescents do not perceive adults or institutions as safe, responsive, and discreet. Thus, prevention efforts should not focus only on increasing knowledge, but also on strengthening trust, disclosure pathways, and supportive school climates [65, 67–69].

Another important implication concerns programme sustainability. Participants perceived the intervention as feasible for school implementation, particularly if integrated into existing structures such as guidance and counselling sessions, class-based activities, or other scheduled school forums. They also suggested that the programme would be more effective if delivered in interactive, discussion-based, and visually engaging formats, rather than through text-heavy or purely didactic approaches. This reinforces the idea that sustainability depends not only on the value of the content, but also on how well the delivery model aligns with adolescents’ learning preferences and with the practical realities of the school setting [70–75].

Taken together, the results suggest that the digital intervention provided an observable additional benefit for knowledge and awareness but not for attitude during the short follow-up period. The distinction between statistical significance and effect magnitude is important: knowledge demonstrated a moderate effect, while awareness showed only a small effect despite remaining significant after multiplicity adjustment. Thus, the intervention appears particularly suitable for strengthening adolescents’ immediate cognitive understanding and recognition of TFSV risks, whereas bigger attitudinal change may require a longer, more interactive, and system-supported intervention [76]. However, the findings also indicate that longer-term attitudinal and behavioural change is likely to depend on a wider network of support that includes families, peers, and trusted school personnel [77, 78]. School-based TFSV prevention may therefore be most effective when digital education is combined with supportive relational environments, repeated reinforcement, and institutional mechanisms that enable adolescents to seek help safely and confidently [79, 80].

### Strengths and limitations

A major strength of this study is the use of a mixed-methods approach, which enabled a more comprehensive evaluation of the digital TFSV prevention education programme than would have been possible using quantitative or qualitative data alone. The quantitative findings demonstrated measurable changes in knowledge, awareness, and attitudes, while the FGD data clarified how participants interpreted the intervention, why some outcomes changed more strongly than others, and what contextual factors influenced engagement and perceived usefulness.

A further strength is that the intervention was delivered in a format that was developmentally and contextually appropriate for adolescents. Participants’ responses suggested that clear explanations, relatable scenarios, and audiovisual delivery increased acceptability and engagement. This indicates that adolescent-friendly digital approaches may be a useful strategy for strengthening TFSV prevention education in school settings.

Several limitations should be considered. First, the quasi-experimental design and the use of only one school per study condition limit causal inference, because intervention allocation could not be separated from unmeasured school-level characteristics. In addition, the intervention group received substantially greater educational exposure than the control group. Although both groups received the standard school-based programme, the intervention group also received six videos through classroom sessions, Zoom meetings, and Instagram. Consequently, the observed differences may reflect not only the intervention content and delivery format, but also greater contact time, repeated exposure, and attention.

Second, engagement with the digital components was incomplete. Not all participants attended the Zoom sessions, and interaction with the Instagram component was limited. This variation in exposure may have affected intervention fidelity and contributed to differences in individual responsiveness. The study also assessed only short-term outcomes, and the between-group analysis was based on post-test score distributions rather than baseline-adjusted estimates of change. The results should therefore be interpreted as post-intervention group differences rather than definitive evidence of causal intervention effects.

Third, the knowledge scale had an internal consistency coefficient of 0.652, below the conventional threshold of 0.70, which may have reduced measurement precision. Awareness and attitude scores were also concentrated near the upper end of their scales, suggesting possible ceiling effects. Furthermore, all outcomes were self-reported and may have been influenced by recall and social desirability bias.

Finally, the qualitative findings were derived from a purposively selected subgroup from one intervention school. Although selection considered variation in scores, attendance, and programme engagement, the FGD may not have represented participants who declined, were unable to attend, or engaged minimally with the intervention. The inclusion of only female adolescents from two public schools in one city also limits transferability to other genders, school settings, and sociocultural contexts.

The study was also not prospectively registered in a public trial registry, although ethical approval and institutional registration were obtained before data collection. Future studies should use multicentre randomized or cluster-randomized designs, baseline-adjusted analyses, longer follow-up, broader populations, and more sensitive outcome measures.

### Implications for nursing practice

These findings have several implications for nursing practice, particularly for school nurses, community nurses, and nurses involved in adolescent health promotion. Nurses can play an important educational role in TFSV prevention by delivering age-appropriate, structured, and digitally relevant education that strengthens adolescents’ knowledge and awareness of online sexual risks. Such education should go beyond general warnings and include concrete examples, relatable scenarios, and practical strategies for recognising and responding to unsafe digital interactions. The findings also suggest that education alone is not sufficient; nurses need to help build trust, confidentiality, and psychologically safe pathways for disclosure and referral so that adolescents feel able to seek help when needed.

The findings further indicate that nursing practice should extend beyond the individual level to include families and school systems. Parental openness, peer support, and trust in school-based support mechanisms appear to influence whether adolescents translate awareness into action. Nurses are therefore well positioned to strengthen TFSV prevention through parent engagement, collaboration with teachers and counsellors, and the development of trauma-informed, adolescent-friendly support systems. In addition, the intervention model suggests that nurse-supported digital education could be integrated into routine school health promotion through engaging audiovisual materials, facilitated discussion, repeated reinforcement, and clear referral pathways.

## Conclusion

This study found that the digital TFSV prevention education programme was associated with higher post-test knowledge and awareness scores than the standard school-based programme. The qualitative findings indicated that relevant content, relatable scenarios, and audiovisual delivery may have supported adolescents’ understanding and recognition of digital sexual risks. However, no additional effect on attitude was demonstrated, as attitude scores increased within both groups and did not differ significantly between groups at post-test. The findings should be interpreted cautiously because one school represented each study condition, intervention exposure differed between groups, engagement with the digital components was incomplete, and the analysis did not estimate baseline-adjusted change. Further multicentre studies with longer follow-up are needed to determine whether improvements in knowledge and awareness translate into sustained protective attitudes and behaviours.

## Supplementary Information

Supplementary Material 1.

## Data Availability

The datasets generated and/or analyzed during the current study are not publicly available due to the sensitive nature of the research topic and the involvement of adolescent participants, but are available from the corresponding author upon reasonable request and subject to appropriate ethical approval.

## Abbreviations

CVI: Content Validity Index
FGDs: Focus group discussions
TFSV: Technology-facilitated sexual violence
