# Supplementary material for: Effectiveness of a school-based educational video intervention for the prevention of technology-facilitated sexual violence among adolescent girls: a sequential explanatory mixed-methods study
Source: BMC Public Health. 2026 Aug 1;26:3297. doi: 10.1186/s12889-026-28700-7 (PMC13749861; doi:10.1186/s12889-026-28700-7)
Supplement: Supplementary file 1 [file 12889_2026_28700_MOESM1_ESM.docx]

**KNOWLEDGE ON TECHNOLOGY-FACILITATED SEXUAL VIOLENCE QUESTIONNAIRE**

This questionnaire is designed to assess your understanding of safety and sexual violence in

the digital world. We will keep your data and responses confidential.

| **No** | **Question** |
| --- | --- |
|  |  |
| 1. | Technology-Facilitated Sexual Violence (TFSV) refers to sexual violence that occurs through digital media, such as chat platforms or social media.  a. True  b. False |
|  |  |
|  |  |
|  |  |
| 2. | TFSV can only occur if the perpetrator and the victim meet in person.  a. True  b. False |
|  |  |
|  |  |
|  |  |
| 3. | Recording and disseminating another person’s private content without their consent constitutes a form of TFSV.  a. True  b. False |
|  |  |
|  |  |
|  |  |
| 4. | Only people we just met on social media can manipulate our emotions (online harassment) as the first step to committing TFSV.  a. True  b. False |
|  |  |
|  |  |
|  |  |
| 5. | Even without physical contact, victims of TFSV can experience prolonged stress.  a. True  b. False |
|  |  |
|  |  |
|  |  |
| 6. | Stalking someone else’s social media accounts constitutes a violation of the law on sexual violence.  a. True  b. False |
|  |  |
|  |  |
|  |  |
| 7. | A boyfriend has the right to access our personal content.  a. True  b. False |
|  |  |
|  |  |
|  |  |
| 8. | TFSV cases often occur because the victim seeks validation on social media, so it is entirely the victim’s fault.  a. True  b. False |
|  |  |
|  |  |
|  |  |
| 9 | Saving digital evidence such as messages or screenshots is important in the event of a TFSV.  a. True  b. False |
|  |  |
|  |  |
|  |  |
| 10 | Victims of TFSV have the right to seek help and report what happened to them.  a. True  b. False |
|  |  |
|  |  |
|  |  |

Choose the answer you think is correct

**RISK AWARENESS ON TECHNOLOGY-FACILITATED SEXUAL VIOLENCE QUESTIONNAIRE**

General Instructions

This questionnaire does not ask about personal experiences.

Please answer based on your own opinions and understanding.

Response Scale (Likert 1–5)

1 = Not at all aware

2 = Not aware

3 = Somewhat aware

4 = Aware

5 = Very aware

| **No** | **Statement/ Question** | **Answer** |
| --- | --- | --- |
|  |  |  |
| 1. | A friend of yours has just broken up with their partner. Their partner is upset about it and is threatening to post their intimate videos on social media.  How aware are you that this situation could lead to TFSV? | 1-5 |
|  |  |  |
|  |  |  |
| 2. | Your friend and her boyfriend engaged in inappropriate behavior on school grounds when no one was around. Another friend of yours recorded the incident without their consent.  To what extent are you aware that this constitutes TFSV? | 1-5 |
|  |  |  |
|  |  |  |
|  |  |  |
| 3. | You met an older person on Instagram. You felt comfortable enough to start dating them, and eventually, out of affection, you agreed to their request to dress more revealingly, even though you had your doubts at first.  How aware were you that this person was a perpetrator of TFSV? | 1-5 |
|  |  |  |
|  |  |  |
|  |  |  |
| 4. | You and your friends have received a message from an unknown number containing a video with sexual content.  If you forward that message, how likely is it that TFSV will occur? | 1-5 |
|  |  |  |
|  |  |  |
| 5. | Regarding risky dating behaviors such as kissing, neck-kissing, physical contact that does not involve sexual intercourse (petting), sexual intercourse, talking on the phone until falling asleep (sleep calls), and sending sensual or sexually suggestive photos (sensual photos).  In your opinion, to what extent can these behaviors lead to TFSV? | 1-5 |
|  |  |  |
|  |  |  |
|  |  |  |
| 6. | I realize that I need to be able to say “no” even when under pressure from my boyfriend or friends. | 1 -5 |
|  |  |  |
| 7. | I realize that keeping my data and social media accounts private is important. | 1 -5 |
| 8. | I am aware that I have the right to refuse requests for photos, videos, or personal information online. | 1 -5 |
|  |  |  |
| 9 | I realize that keeping digital evidence is a way to protect myself. | 1 -5 |
| 10 | If I experience TFSV, I should seek professional help from a guidance counselor, peer counselor, etc. | 1 -5 |
|  |  |  |

**ATTITUDE TOWARD THE PREVENTION OF TECHNOLOGY-FACILITATED SEXUAL VIOLENCE (TFSV) QUESTIONNAIRE**

General Instructions

for adolescent female participants

Please answer based on your opinion regarding the following statements.

This questionnaire uses a Likert scale, with scores ranging from 1 to 5.

1 = Strongly disagree,

2 = Disagree,

3 = Undecided,

4 = Agree,

5 = Strongly agree.

| **No** | **Statement** |
| --- | --- |
|  |  |
| **A** | **Attitudes Toward Awareness and Risk of TFSV** |
| 1. | I realize that knowledge about sexuality can be gained through digital media  . |
| 2. | Sending personal photos or videos can be risky if they are misused. |
| **B** | **Attitudes Toward Prevention and Self-Protection** |
| 3. | I need to be careful when communicating with people I’ve just met online. |
| 4. | Managing the security and privacy of my digital accounts is my own responsibility. |
| **C** | **Attitudes Toward Norms, Consent, and Boundaries** |
| 5. | Pressure from a boyfriend, girlfriend, or friend to send personal content is never acceptable. |
| 6. | I set boundaries in my digital interactions with everyone. |
| **D** | **Attitudes Toward Reporting and Seeking Help** |
| 7. | Victims of TFSV should not be blamed for what they have experienced. |
| 8. | Schools and health services need to be involved in addressing TFSV. |
| **E** | **Attitudes Toward Empathy & Social Support** |
| 9. | I won’t share personal information with others, even as a joke. |
| 10. | Preventing TFSV is a shared responsibility, not just that of the victims. |
